# Connecting the Global Cancer Community

**DOI:** 10.1200/JGO.2015.001503

**Published:** 2015-10-28

**Authors:** David J. Kerr

**Affiliations:** University of Oxford, Oxford, United Kingdom

No man is an island, entire of itself; every man is a piece of the continent, a part of the main. If a clod be washed away by the sea, Europe is the less, as well as if a promontory were, as well as if a manor of thy friend's or of thine own were: any man's death diminishes me, because I am involved in mankind, and therefore never send to know for whom the bell tolls; it tolls for thee.

John Donne (1572-1631) was one of the leading metaphysical poets of the Renaissance and one of his writings captures two themes that resonate with us to this day: the connectedness of mankind and the absolute likelihood that some day, everyone you know will die. Without dwelling on the latter, the world is more interconnected now than at any stage in its history, geopolitically, economically, electronically, culturally, intellectually and, as we now know, genetically.

Guy Murcia says that we're all family. You have at least a million relatives as close as tenth cousin, and no one on Earth is further removed than your fiftieth cousin. Murcia also describes our kinship through an analysis of how deeply we share the air. With each breath, you take 10 sextillion atoms into your body and, owing to the wind's ceaseless circulation, over a year's time you have intimate relations with oxygen molecules exhaled by every person alive as well as everyone who ever lived.^1^

This is why the *Journal of Global Oncology* (*JGO*) is important. It aims to give a voice and a home to publications from health care workers, academics, policy leaders, epidemiologists, and public health specialists who seek to improve cancer control in economically challenged countries and economically challenged communities embedded in wealthier nations. What is meant by economically challenged? Given the controversy surrounding the term “developing countries” and confusion over its definition, it may be simpler to resort to the arithmetic of the World Bank, which classifies countries into four income groups set each year on July 1. Economies were divided according to 2011 gross national income per capita by using the following ranges of income^2^: low-income countries, US $1,045 or less; lower-middle-income countries, between US $1,045 to US $4,125; upper-middle-income countries, between US $4,126 and US $12,476; and high-income countries, above US $12,476. To put this into perspective, it has been estimated that the health minister of Kenya has $10 per capita to spend on all health care in this populous East African nation. This redefines and throws into sharp contrast the sort of debate we have in the West and in the North about rationing health resources, especially in oncology, as we seek but often fail to find value in many of the new therapeutic agents being approved, with costs in the tens of thousands of dollars.^3–5^ Clearly these issues are equally applicable to populations in rural Appalachia and on American Indian reservations in the high-income United States. The scope of *JGO* encompasses research into cancer control in each of these seemingly disparate populations.

Despite the often rudimentary research infrastructure, oncologists working in poor countries are eager to publish their work and contribute to the wider global community, reflecting the disease burden germane to their locale or differential response to drugs in patients who have different comorbidities than patients in the West, and explore comparative cancer biology in regions which paleontologists consider may have been the bath of birth. Thus, we may generate new knowledge by the systematic study of ethnic groups, enlarge the patient pool to speed clinical trials, and widen international effort on basic and translational research. It will have an inductive effect on training young oncologists who will have to learn the basics of clinical and translational research and the disciplines of publishing in international journals.

Why should well established, well-resourced oncologists read *JGO*? One reason is the prospect of learning something new. Indefatigable oncologists from low-income countries and their drive to find cost-effective solutions for their patients can re-energize colleagues from the North and may generate a desire in these oncologists to contribute personally to support the global community through volunteerism or some other ingenuously lateral ideas aimed at treating cancer for a dollar a day.^6^ Muir Gray and Kerr^7^ describe three health care revolutions: the nineteenth century saw an extraordinary improvement in public health. The second health care revolution in the twentieth century saw the application of knowledge, and the third health care revolution in the twenty-first century will be driven by equity and implementation—knowledge into action. Perhaps *JGO* can help support the third health care revolution by engendering a global drive toward equity of cancer control.

*JGO* has been invited by its distinguished editorial board to campaign on issues of global oncology. The Oxford English Dictionary (to which all of us should subscribe!) defines a campaign as an organized course of action to achieve a goal and defines activism as the policy or action of using vigorous campaigning to bring about political or social change. Given the fantastic resources of the American Society of Clinical Oncology, Union for International Cancer Control, and European Society for Medical Oncology, along with committed leadership, talented membership, educational content, policy expertise, and a genuine desire to reach out to underserved communities of patients with cancer, there is a great opportunity to get behind a few of the key issues highlighted by oncologists in low-income countries (eg, raising awareness, earlier detection of disease) and change the world.

Nothing less.

“The fundamental delusion of humanity is to suppose that I am here and you are out there.” Yasutani Roshi, Zen master (1885-1973).

“Woven into our lives is the very fire from the stars and genes from the sea creatures, and everyone, utterly everyone, is kin in the radiant tapestry of being.” Elizabeth A. Johnson, *Women, Earth, and Creator Spirit*.
